# DEPROMP Trial: the additive value of PSMA-PET/CT-guided biopsy for prostate cancer management in biopsy naïve men—study protocol for a randomized trial

**DOI:** 10.1186/s13063-023-07197-0

**Published:** 2023-03-06

**Authors:** P. Krausewitz, R. A. Bundschuh, F. C. Gaertner, M. Essler, U. Attenberger, J. Luetkens, G. Kristiansen, M. Muders, C-H. Ohlmann, S. Hauser, J. Ellinger, M. Ritter

**Affiliations:** 1 Department of Urology and Pediatric Urology, University Hospital Bonn, Bonn, Germany; 2 Department of Nuclear Medicine, University Hospital Bonn, Bonn, Germany; 3 Department of Diagnostic and Interventional Radiology, University Hospital Bonn, Bonn, Germany; 4 Institute of Pathology, University Hospital Bonn, Bonn, Germany; 5 Department of Urology, Johanniter Hospital Bonn, Bonn, Germany

**Keywords:** PSMA-PET/CT, Prostate biopsy, Management plan, Clinically significant prostate cancer, Multiparametric MRI

## Abstract

**Background:**

The primary objective is to determine the proportion of men with suspected prostate cancer (PCA) in whom the management plans are changed by additive gallium-68 prostate-specific membrane antigen positron emission tomography/computed tomography (PSMA-PET/CT) guided prostate biopsy (PET-TB) in combination with standard of care (SOC) using systematic (SB) and multiparametric magnetic resonance imaging-guided biopsy (MR-TB) compared with SOC alone. The major secondary objectives are to determine the additive value of the combined approach of SB + MR-TB + PET-TB (PET/MR-TB) for detecting clinically significant PCA (csPCA) compared to SOC; to determine sensitivity, specificity, positive and negative predictive value and diagnostic accuracy of imaging techniques, respective imaging classification systems, and each biopsy method; and to compare preoperatively defined tumor burden and biomarker expression and pathological tumor extent in prostate specimens.

**Methods:**

The DEPROMP study is a prospective, open-label, interventional investigator-initiated trial. Risk stratification and management plans after PET/MR-TB are conducted randomized and blinded by different evaluation teams of experienced urologists based on histopathological analysis and imaging information: one including all results of the PET/MR-TB and one excluding the additional information gained by PSMA-PET/CT guided biopsy. The power calculation was centered on pilot data, and we will recruit up to 230 biopsy-naïve men who will undergo PET/MR-TB for suspected PCA. Conduct and reporting of MRI and PSMA-PET/CT will be performed in a blinded fashion.

**Discussion:**

The DEPROMP Trial will be the first to evaluate the clinically relevant effects of the use of PSMA-PET/CT in patients with suspected PCA compared to current SOC. The study will provide prospective data to determine the diagnostic yields of additional PET-TB in men with suspected PCA and the impact on treatment plans in terms of intra- and intermodal changes. The results will allow a comparative analysis of risk stratification by each biopsy method, including a performance analysis of the corresponding rating systems. This will reveal potential intermethod and pre- and postoperative discordances of tumor stage and grading, providing the opportunity to critically assess the need for multiple biopsies.

**Trial registration:**

German Clinical Study Register DRKS 00024134. Registered on 26 January 2021.

**Supplementary Information:**

The online version contains supplementary material available at 10.1186/s13063-023-07197-0.

## Administrative information

Note: The numbers in curly brackets in this protocol refer to the SPIRIT checklist item numbers. The order of the items has been modified to group similar items (see http://www.equator-network.org/reporting-guidelines/spirit-2013-statement-defining-standard-protocol-items-for-clinical-trials/).

**Table:** 

Title {1}	DEPROMP Trial: the additive value of PSMA-PET/CT-guided biopsy for prostate cancer management in biopsy naïve men—study protocol for a randomized trial
Trial registration {2a and 2b}.	DRKS 00,024,134, German Clinical Study Register, January 26, 2021 www.drks.de/drks_web/navigate.do?navigationId=trial.HTML&TRIAL_ID=DRKS00024134
Protocol version {3}	approved protocol version 4.0 and patient information 3.1, May 17, 2022
Funding {4}	The Commission of Clinical Studies of the Medical Faculty of the Rheinische Friedrich-Wilhelms-University Bonn and the University Hospital Bonn, Department of Urology, are funding the study (process number 2019-FKS-07, study code URO-201901-DEPROMP).
Author details {5a}	(1) University Hospital Bonn, Department of Urology and Pediatric Urology, Bonn, Germany (2) University Hospital Bonn, Department of Nuclear Medicine, Bonn, Germany (3) University Hospital Bonn, Department of Diagnostic and Interventional Radiology, Bonn, Germany (4) University Hospital Bonn, Institute of Pathology, Bonn, Germany (5) Johanniter Hospital Bonn, Department of Urology, Bonn, Germany
Name and contact information for the trial sponsor {5b}	Sponsor contact: University Hospital Bonn, Germany Principal Investigator contact: Philipp Krausewitz, M.D. Department of Urology and Pediatric Urology, University Hospital Bonn Venusberg Campus 1, 53,127 Bonn, Germany Email: Philipp.Krausewitz@ukbonn.de Tel.: + 49 151 18,853,551 Orcid-ID 0000–0002-8213–9975
Role of sponsor {5c}	There are no disclose contractual agreements with sponsors or funders that limit access for investigators for collection, management, analysis, and interpretation of the data; writing of the report; and decision for publication. The investigators have ultimate authority over any of these activities.

## Introduction

### Background and rationale {6a}

Prostate cancer (PCA) is biologically characterized by strong inter- and intratumoral heterogeneity, making it challenging to differentiate indolent cancer (International Society of Urological Pathology (ISUP) grade 1 PCA, nsPCA) from potentially lethal, clinically significant carcinomas (≥ ISUP grade 2 PCA, csPCA). PCA and its treatment affect patients’ quality of life in several areas [1, 2].

Increasing individualized therapy strategies drive the importance of reliable risk stratification during initial staging to adjust clinical management plans. Multiparametric magnetic resonance imaging of the prostate (MRI) has improved the detection of csPCA with a sensitivity of 88% and beneficially reduced the detection of nsPCA [3]. Guidelines recommend MRI before prostate biopsy, and its use has steadily increased worldwide [4]. Nevertheless, the detection accuracy of the MRI depends on cancer differentiation and is limited by a rather low PPV of 34–65% [3, 5]. Hence, the debate persists about whether MRI-targeted biopsy (MR-TB) should be used in place of systematic biopsy (SB) or in conjunction with it. Despite the higher detection of csPCA by the combined approach, a sampling error in one-third of patients is still to be expected [6]. Since morbidity-associated, cost-intensive diagnostic, and therapeutic measures are based upon biopsy results, there is a need to optimize the biopsy pathway.

Additional imaging and/or biomarkers could improve risk stratification. The prostate-specific membrane antigen (PSMA) seems to be both a promising biomarker and a key element for improved imaging for PCA detection. Its expression is associated with high-grade cancer, increased tumor progression, and the risk of early biochemical recurrence [7, 8]. Today, gallium-68 PSMA positron emission tomography/computed tomography (PSMA-PET/CT) is primarily used in cases of biochemical recurrence and for primary staging of high-risk patients and has limited availability [9, 10]. However, based on its high diagnostic accuracy, clinical utility, and recent approval by the US Food and Drug Administration for the detection of PCA, PSMA-PET/CT is expected to contribute significantly to the diagnostic process of primary PCA in the future.

Several recent series have already demonstrated improved intraprostatic diagnostic accuracy for PCA using PSMA-PET/CT compared to MRI [11–13] and the utility of PSMA-PET/CT-guided fusion biopsy (PET-TB) to optimize PCA detection [14, 15]. The combined approach of PSMA-PET/CT and MRI results in improved csPCA detection with enhanced NPV of 91% in biopsy-naïve patients [16] and an optimized description of local tumor extension, both crucial for the management planning [17]. Moreover, a significant impact on treatment strategies by PSMA-PET/CT was shown for PCA-proven men, resulting in intra- and inter-modal adjustments of management in up to 75% of men [18] and an optimized discrimination between unifocal, multifocal, and oligometastatic disease, decisive for individualized targeted and multimodal PCA-management [19, 20]. However, the gain in information through optimized initial staging raises previously unanswered questions: Which findings result in treatment changes and which patients benefit from altered therapy management?

Valid study results on these questions are currently not available. The DEtection rate of clinically significant PROstate cancer by mpMRI and PSMA-PET/CT fusion biopsy (DEPROMP) trial aims to determine the benefit of additive PET-TB and its impact on the diagnostic and therapeutic algorithm of PCA patients. Hereby, the combined approach of SB, MR-TB, and PET-TB (PET/MR-TB) has the potential to increase the detection of csCPA, improve the assessment of tumor extent and stage, and subsequently ameliorate the management of PCA patients significantly.

### Objectives {7}

The DEPROMP Trial aims to determine the clinically relevant effects of the additive use of PSMA-PET/CT in biopsy-naïve patients with suspected PCA compared to current SOC. In particular, its influence on medical treatment planning and on the detection of csPCA is investigated. A detailed explanation of the study objectives is presented in Table 1.

**Table 1:** Study objectives

**Primary objective**
➢ To determine the proportion of patients in whom management plans are changed by additional PET-TB compared to the SOC using SB and MR-TB
**Secondary study objectives**
➢ To determine the additive value of PET/MR-TB for detecting csPCA in men undergoing initial biopsy for suspected PCA compared to SOC
➢ To determine sensitivity, specificity, PPV, NPV, and diagnostic accuracy for PCA and csPCA of MRI, PSMA-PET/CT, biopsy modalities (SB vs. MR-TB vs. PET-TB vs. PET/MR-TB), and the respective imaging classifications (PIRADS V2.1 classification, PROMISE miTNM classification V1.0, miPSMA score)
➢To determine the additive value of more than one targeted biopsy per MRI- or PSMA-PET/CT suspicious lesion
➢ To determine the concordance of tumor burden defined preoperatively by MRI and PSMA-PET/CT and tumor extent in the prostatectomy specimen using whole-mount sections
➢ To determine the frequencies of predefined adverse events CTCAE grade 3 in the context of PET/MR-TB in relation to the total number of biopsy cores taken and anticoagulants intake
➢ To determine the concordance of biomarker expression (PTEN and PSMA) between tumor-bearing prostate specimens (biopsy cores vs. prostatectomy specimens) as well as concordance between PSMA expression of PSMA-PET/CT imaging and prostatectomy specimens and tumorous lymph nodes

*PET* Positron-emission tomography, *PET-TB* ultrasound-guided PSMA-PET/CT fusion biopsy, *SOC* Standard of care, *SB* Systematic 12-core ultrasound biopsy of the prostate, *MR-TB* ultrasound-guided MRI fusion biopsy, *PET/MR-TB* combined SB + MR-TB + PET-TB, *PCA* Prostate cancer, *csPCA* clinically significant prostate cancer, defined as PCA with Gleason score ≥ 7a, *PPV* Positive predictive value, *NPV* Negative predictive value, *MRI* Multiparametric magnetic resonance imaging, *PI-RADS* Prostate Imaging-Reporting and Data System, *PSMA* Prostate-specific membrane antigen, *PROMISE* Prostate Cancer Molecular Imaging Standardized Evaluation, *miTNM* molecular imaging TNM system, *miPSMA score* molecular imaging PSMA expression score, *CTCAE* Common Terminology Criteria for Adverse Events

### Trial design {8}

The DEPROMP Trial is a prospective, interventional, exploratory investigator-initiated, single-group, cohort assessment study. After all patients have undergone PET/MR-TB, risk stratification and treatment planning are compared in a randomized, blinded fashion between the SOC (SB + MR-TB) and PET/MR-TB groups.

## Methods: participants, interventions, and outcomes

### Study setting {9}

Patients are referred to the study centers (University Hospital Bonn or the community Clinic Johanniter Hospital Bonn, Germany) because of suspected localized PCA. Suspicion of PCA is based on elevated prostate-specific Antigen (PSA) > 4 ng/ml, abnormal digital rectal examination (DRE), and/or abnormal findings on transrectal ultrasound (US).

### Eligibility criteria {10}

Patients will be screened for eligibility to participate using the inclusion and exclusion criteria listed in Table 2.

**Table 2:** Inclusion and exclusion criteria

**Inclusion criteria**
1. Men suspected localized PCA based on at least one of the following criteria:
(a) Repeated PSA value ≥ 4 ng/ml
(b) Suspicious palpation of the prostate on digital rectal examination
(c) Tumor-suspicious findings on transrectal ultrasound
2. Age ≥ 45 and < 76 years
3. No previous prostate biopsy
4. Ability to give written informed consent, participate in and comply with study
**Exclusion criteria**
1. Allergy to the radiopharmaceutical 68 Ga-PSMA or to preparations with similar chemical structure
2. Abuse of medications, drugs, or alcohol
3. PSA level elevation > 100 ng/ml with resulting suspected advanced metastatic PCA
4. Chronic ongoing severe renal disease defined by estimated creatinine clearance < 30 ml/min

*PCA* Prostate cancer, *PSA* Prostate-specific antigen, *PSMA* Prostate-specific membrane antigen

### Who will take informed consent? {26a}

If eligible, patients will receive information sheets. Physicians of the study group will discuss the trial with patients in light of the information provided. Patients will then be able to have an informed discussion with the participating consultant. Physicians will obtain written informed consent from patients willing to participate in the trial.

### Interventions

#### Explanation for the choice of comparators {6b}

Diagnosis and risk stratification will be based on histopathological analysis and imaging information. In the control group, SOC biopsy results without PET-TB information are presented to the reviewers before they propose a treatment plan, whereas in the intervention group, all PET/MR-TB results are presented to the reviewers before they establish the treatment plan. Changes in therapeutic management include not only radical changes (localized therapy versus systemic therapy) but also variations in the local management. Therefore, study-relevant management changes are defined as any variation caused by the additive PSMA-PET/CT, including inter- and intra-modal adjustments, e.g., nerve-sparing approach or extent of lymph node dissection.

#### Intervention description {11a}

All patients will be investigated for prostate cancer by DRE, transrectal US, MRI, SB, MR-TB (standard of care), and additional PSMA-PET/CT and PET-TB.

#### Visit 1

In all patients, DRE and transrectal US will be performed. Moreover, Eastern Cooperative Oncology Group performance status, concomitant disease, and medication are assessed, and a PSA/free PSA will be determined.

#### Visit 2

##### MRI

**Fig. 1:**
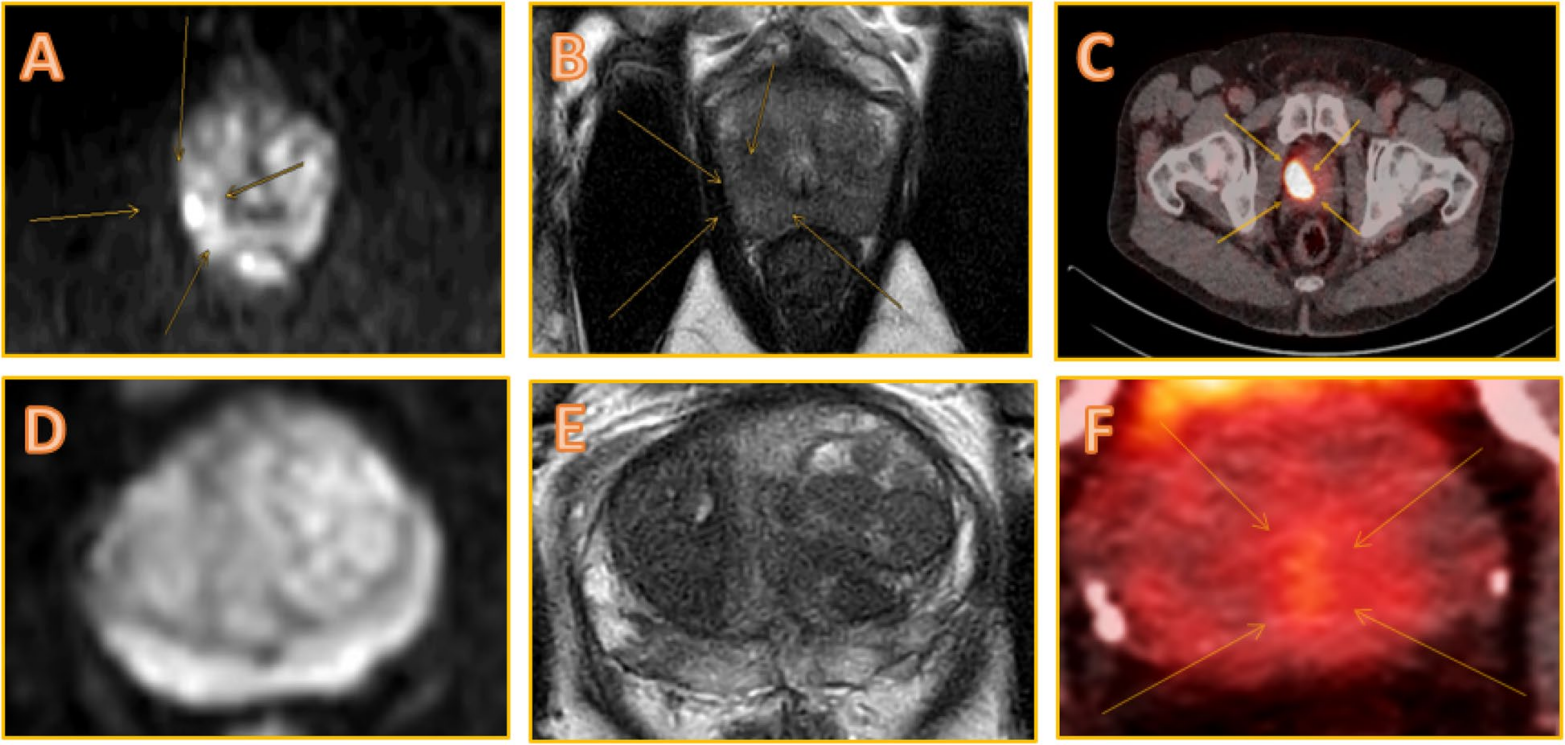
Annotation of suspicious lesions on imaging. **A**–**C** Concordant positive findings of clinically significant prostate cancer (csPCA) in the peripheral zone of the right prostate lobe on multiparametric magnetic resonance imaging (MRI) and gallium-68 prostate-specific membrane antigen positron emission tomography/computed tomography (PSMA-PET/CT). In the DEPROMP Trial, uro-radiologists (**A**, **B**) and nuclear medicine physicians (**C**) annotate defined suspicious lesions in imaging layers to enable effective and clear interdisciplinary communication before targeted biopsy (TB) as demonstrated in exemplary fashion. An example of a false-negative MRI with true-positive PSMA-PET/CT in the posterior transitional zone of the left prostate lobe is shown in series **D**–**F**. The PSMA-PET/CT-guided TB detected a solitary, Gleason 3 + 4 PCA. In both cases, radical prostatectomy confirmed the risk stratification based on biopsy yields

Imaging will be conducted as the standard of care using the Prostate Imaging Reporting and Data System (PI-RADS) in all patients. Acquisition protocol includes T2-weighted sequences in transverse, coronal, and sagittal planes; diffusion-weighted imaging; and dynamic contrast-enhanced T1-weighted perfusion sequences. Two expert uro-radiologists on-site rate and report the MRI results according to PI-RADS v2.1. [21]; ≥ 3 PI-RADS-rated lesions are described including location, size, dynamic contrast enhancement, and ADC values. Furthermore, suspicious lesions are graphically annotated in the MRI sequences (Fig. 1), and suspicious lymph nodes and bone metastasis are reported. Capsular infiltration and extraprostatic extension will also be determined. The reporting uro-radiologists will be blinded to the corresponding PSMA-PET/CT report to avoid bias.

##### PSMA-PET/CT

Imaging is performed at the Department of Nuclear Medicine of the University Hospital Bonn in all patients. [^68^ Ga]Ga-DKFZ-PSMA-11 (HBED-CC) is prepared on-site in compliance with good laboratory practice guidelines. [^68^ Ga]Ga-DKFZ-PSMA-11 will be administered based on patient weight (2 MBq/kg). After an adequate incubation time (optimal range: 50–100 min), PSMA-PET examination and non-contrast-enhanced low-dose CT are performed. Two highly experienced nuclear medicine physicians at the study site rate PSMA-PET/CTs for each patient and lesion using the PROMISE miTNM V1.0 evaluation system published by Eiber et al. [22]. According to PROMISE miTNM V1.0, intraprostatic lesions are visually graded in terms of PSMA expression by miPSMA expression scores 0 (“none,” uptake below blood pool), 1 (“low,” uptake equal to or higher than blood pool, but less than liver), 2 (“intermediate,” uptake equal to or higher than liver, but less than parotid gland), and 3 (“high,” uptake equal to or higher than parotid gland). Intraprostatic lesions with an miPSMA expression score ≥ 2 (uptake equal to or higher than the liver) are rated as positive for PCA [22]. Lesions rated with an miPSMA expression score of 1 are defined as equivocal findings. Intraprostatic extension and location of targetable lesions (miPSMA ≥ 1) are reported in detail by annotation (Fig. 1). For effective and clear interdisciplinary communication, PSMA-PET/CT findings will be additionally reported using a study-specific map derived from the PIRADS V2.1 prostate sector map [21]. In addition, the maximum standardized uptake value (SUVmax) per suspicious lesion is collected, and suspicious lymph nodes and bone metastasis as well as capsular infiltration and extraprostatic extension are determined. The reporting nuclear medicine radiologists will be blinded to the corresponding MRI report to avoid bias.

#### Visit 3

##### Prostate biopsy

**Fig. 2:**
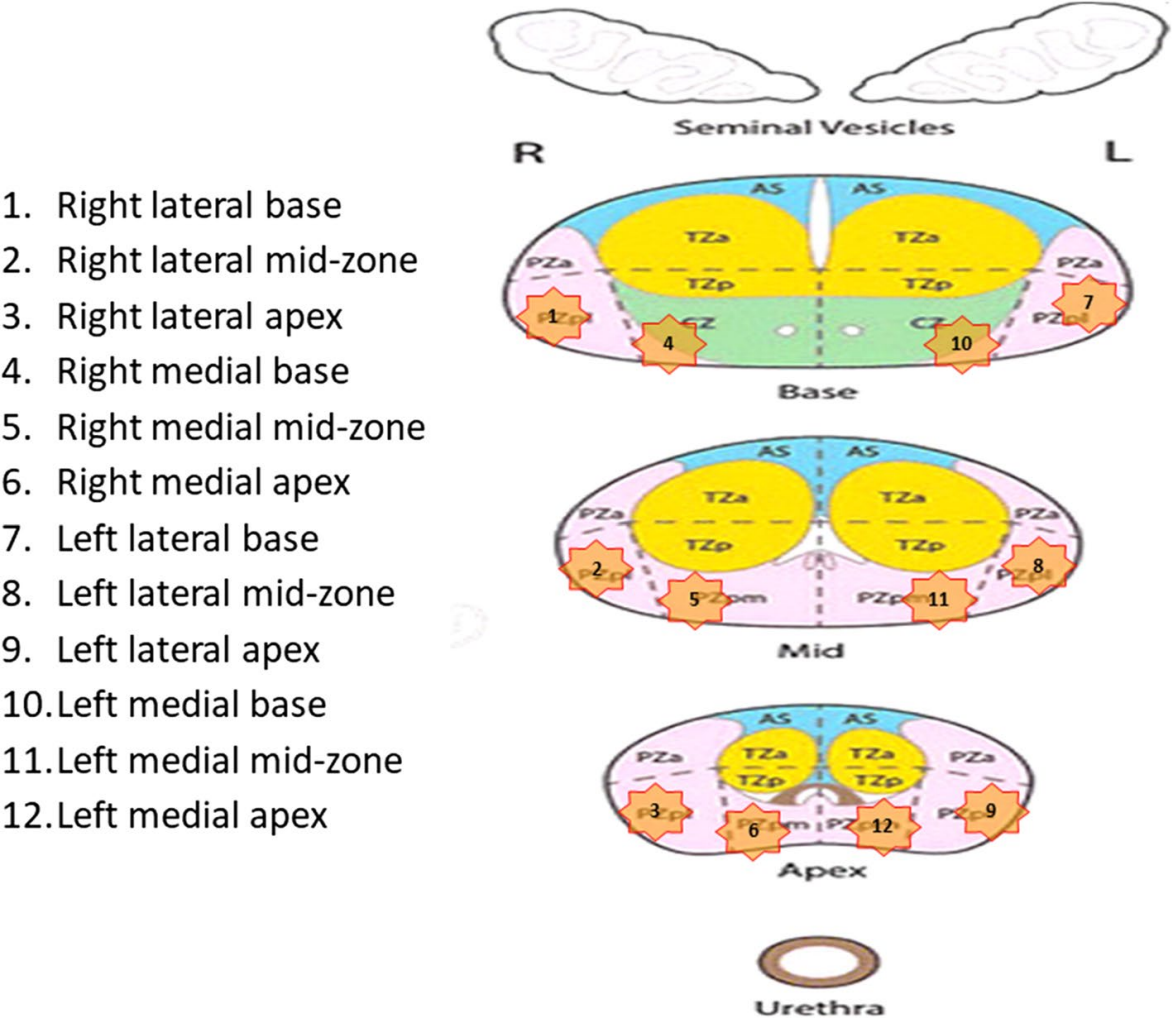
Institutional standardized systematic 12-core prostate biopsy mapping scheme. The standardized scheme for 12-core systematic biopsy (SB) of the prostate used in the DEPROMP Trial. Due to standardized SB, cores 1/2/3/7/8/9 are representative of the areas PZpl, and cores 4/5/6/10/11/12 are representative of the areas CZ and PZpm defined by PIRADS v2.1 sector map [1]

The same physician performs SB and MR- and PET-targeted biopsies in one session. A pre-defined, software-assisted template is used for the institutional standardized biopsy in all participants. Due to standardization, cores 1/2/3/7/8/9 represent the outer peripheral posterolateral zone, and cores 4/5/6/10/11/12 represent the outer peripheral posteromedial zone and the central zone defined by PIRADS v2.1 sector map (Fig. 2). Biopsies will be conducted under antibiotic prophylaxis, rectal cleansing, and local anesthesia. Targeted tissue sampling will be performed from the areas classified as tumor-suspicious by MRI (PI-RADS ≥ 3) and/or PSMA-PET/CT (miPSMA ≥ 1). Up to three identified lesions on PSMA-PET/CT and/or MRI will be targeted with three biopsies per target. Software-assisted fusion technique (KOELIS Trinity®) is used for both SB and image-guided biopsies. Targeted samples are color-coded to allow for subsequent differential analysis. All biopsies are separately documented and sent for histopathological analysis.

##### Histopathology

The analysis will be conducted according to the guidelines [4]. Specifically, specimens are labeled by location and, if applicable, whether they were obtained by SB, PET-TB, or MR-TB. Furthermore, the number of positive cores and the highest Gleason score per prostate lobe, per biopsy method, and per suspicious area (MRI and/or PSMA-PET/CT) are determined. Moreover, the loss of the tumor suppressor gene phosphatase and tensin homolog (PTEN) and PSMA expression will be determined in tumor-bearing biopsy specimens by immunohistochemistry. PSMA expression will be rated by an institutional-established valid semi-quantitative scale ranging from 0 to 3: “none,” “low,” “intermediate,” or “high,” respectively.

#### Visit 4

##### Risk stratification and conduct of management plan

Diagnosis and risk stratification will be based on histopathological analysis and imaging information. To reduce bias from natural variance in clinician recommendations, management plans will be conducted randomized and blinded by four independent specialists using institutional-based questionnaires (Additional file 1: Appendix). Participants will be allocated to either SOC- or SOC and PET-TB group for review. Hence, the reviewers will be presented with either all PET/MR-TB results or SOC biopsy results without PET-TB information. This results in four independently proposed management plans per patient.

#### Visit 5

##### Radical prostatectomy

Depending on the patient’s preference and tumor stage, active therapy (radical prostatovesiculectomy, radiotherapy, multimodal therapy) or active surveillance will be performed. Histopathology of prostatectomy specimens, if available, will be conducted using whole-mount specimens. Pathological tumor extent will be described in detail concerning spatial cancer expansion defined by PI-RADS v2.1 sector map. Tumor stage and grading will be compared to preoperatively defined tumor burden by imaging and biopsy results. In case of active surveillance, re-biopsy will be conducted following the DEPROMP protocol including additive PET-TB and analysis of biomarkers. In addition, a translational, exploratory analysis of biomarker expression in pre- and postoperative staging will be performed. PSMA expression of tumor-bearing prostate specimens (biopsy cores vs. prostatectomy specimens), tumorous lymph nodes, and initial PSMA-PET/CT imaging will be compared.

#### Criteria for discontinuing or modifying allocated interventions {11b}

On participants’ request interventions can be discontinued but not modified. All study data collected up to the point of patient withdrawal will be included in the final analysis.

#### Strategies to improve adherence to interventions {11c}

To improve adherence to intervention protocols, uniform work protocols were defined for radiologic and nuclear imaging, the biopsy procedure, and histopathologic evaluation of biopsy and prostatectomy specimens.

#### Relevant concomitant care permitted or prohibited during the trial {11d}

During the study, there are no restrictions on concomitant treatments and interventions. However, medications that have an anticoagulant effect or an impact on PSA levels will be documented.

#### Provisions for post-trial care {30}

Study-related health injuries are compensated with a maximum coverage of 500.000 euros per participant. The insurance covers possible injuries suffered by the participant directly or indirectly as a result of the use of study-related radiation applications or interventions in connection with the clinical trial.

Patients will be followed up after the biopsy by their urologist or on the study site depending on the patient’s preference for 2 years. PSA levels and PCA-specific diagnostic and therapeutic measures will be recorded quarterly. Afterwards, participants will continue to receive care from their urologist reimbursed by public and private health insurance.

### Outcomes {12}

The impact of PET/MR-TB on management intent is determined randomized by questionnaires evaluation after biopsy has been performed (Additional file 1: Appendix). Changes in management plans are any changes resulting from additional information obtained through PET-TB, including inter- and intra-modal adjustments. Diagnostic accuracy of significant cancer detection is determined using the definitions for csPCA with ISUP grade group ≥ 2. On-site radiologists will grade MRI according to PI-RADS version 2.1 [21]. PSMA-PET/CT images are evaluated per patient and per lesion according to the PROMISE miTNM classification system and the miPSMA expression score at the University Hospital Bonn [22]. PSMA expression will be rated by an established validated semi-quantitative analysis.

#### Primary outcome

The primary outcome is the proportion of men with suspected prostate cancer (PCA) in whom the management plans are changed by additive PSMA-PET/CT-guided prostate biopsy in combination with standard of care using systematic and MRI-guided biopsy compared with SOC alone. Treatment plans are performed randomized for each patient based on information obtained by SOC biopsy or combined PET/MR-TB using questionnaires within 21 days of biopsy. The proposed treatment plans will be compared at intramodal and intermodal levels, and the number of patients with modified treatment regimens will be determined.

#### Secondary outcomes

The following are the secondary outcomes of the study:

- To determine the additive value of PET/MR-TB for detecting csPCA in men undergoing initial biopsy for suspected PCA compared to SOC
- To determine the sensitivity, specificity, PPV, NPV, and diagnostic accuracy for PCA and csPCA of MRI, PSMA-PET/CT, biopsy modalities (SB vs. MR-TB vs. PET-TB vs. PET/MR-TB), and the respective imaging classifications (PIRADS V2.1 classification, PROMISE miTNM classification V1.0, miPSMA score)
- To determine the additive value of more than one targeted biopsy per MRI- or PSMA-PET/CT suspicious lesion
- To determine the concordance of tumor burden defined preoperatively by MRI and PSMA-PET/CT and tumor extent in the prostatectomy specimen using whole-mount sections
- To determine the frequencies of predefined adverse events CTCAE grade 3 in the context of PET/MR-TB in relation to the total number of biopsy cores taken and anticoagulants intake
- To determine the concordance of biomarker expression (PTEN and PSMA) between tumor-bearing prostate specimens (biopsy cores vs. prostatectomy specimens) and concordance between PSMA expression of PSMA-PET/CT imaging and prostatectomy specimens and tumorous lymph nodes

The timing of measurements of outcomes a–c and e will occur after histopathologic analysis of the prostate biopsy and completion of study visit 4 (appropriately defined as study day 21).

The timing of measurements of outcomes d and f will occur after histopathologic analysis of prostatectomy specimens and completion of study visit 5 (appropriately defined as study day 180).

### Participant timeline {13}

**Fig. 3:**
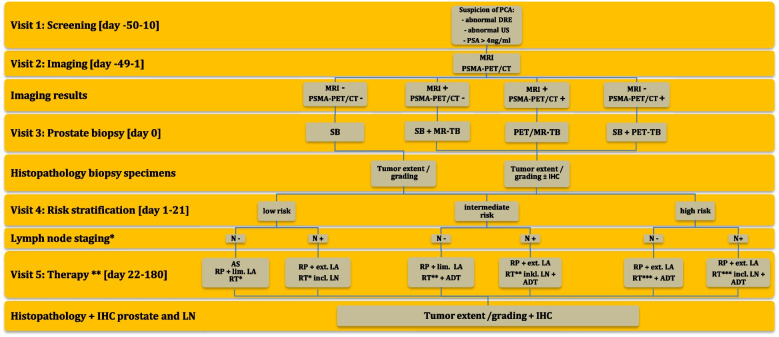
DEPROMP Trial scheme

**Fig. 4:**
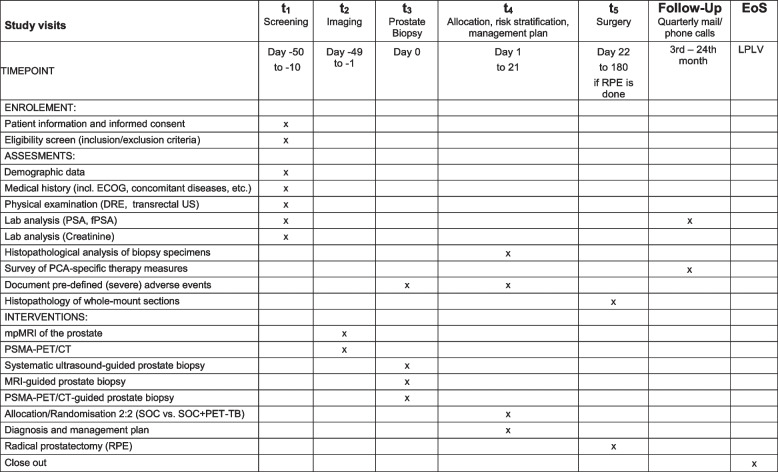
SPIRIT figure of the DEPROMP Trial

Screening for eligibility will be performed on day 50 to 10 days before prostate biopsy. Imaging by transrectal US, MRI, and PSMA-PET/CT will be performed within 1–49 days before the procedure. Histopathology of prostate biopsy specimens and risk stratification (based on imaging results and histopathology) will be performed on days 1–21. If patients are applicable and do request radical prostatectomy, the procedure will be performed risk-adapted within 22–180 days after prostate biopsy. PSA levels will be determined quarterly for 2 years, and PCA-specific diagnostic and therapeutic measures will be recorded. The timeline of the study and assessments are given in Figs. 3 and 4.

### Sample size {14}

There are no published data on treatment plan changes due to the initial diagnostic performance of PET-TB to derive an optimal robust power calculation. However, the power calculation was based on pilot data suggesting inter- and intra-modal PSMA-PET/CT-dependent changes in the management plan after the detection of prostate cancer in the order of 25–75% of patients [18, 23]. Hence, we will recruit up to 230 biopsy-naïve men. The calculation of the sample size of 200 evaluable patients allows us to determine an expected rate of change in the therapeutic approach of 50% with a precision of ± 6.9% defined by the confidence interval. To allow for a dropout rate of 13%, we will recruit 230 patients. Men who drop out of the study prematurely (i.e., before the biopsies at visit 3) will be replaced by additional study participants.

### Recruitment {15}

Patients will be screened for eligibility to participate in a dedicated consultation until the target population is reached. The enrolment period will extend over 24 months.

### Assignment of interventions: allocation

#### Sequence generation {16a}

A computer-based randomization tool generates the allocation sequence and assigns participants’ biopsy results to either the SOC- or SOC and PET-TB group for review. All patients who received prostate biopsy will be randomized without further restrictions.

#### Concealment mechanism {16b}

Allocation concealment will be ensured, as the service will not release the opaque randomization code.

#### Implementation {16c}

A computerized randomization tool generates an opaque allocation sequence. Participants’ biopsy results will be assigned to either SOC or SOC and PET-TB with a 2:2 allocation.

### Assignment of interventions: blinding

#### Who will be blinded {17a}

The reporting uro-radiologists and nuclear medicine physicians are blinded to the corresponding PSMA-PET/CT or MRI results, respectively.

The reporting pathologist may know the technique used for the targeted biopsies. Otherwise, he will not have direct access to MRI or PSMA-PET/CT results.

For risk stratification and the proposal of management plans, two evaluators will be blinded by attenuating the findings of PET-TB for each patient.

#### Procedure for unblinding if needed {17b}

The urologist who performs prostate biopsy will be provided access to all available information to perform targeted biopsies and discuss the findings with patients if indicated. Moreover, a multidisciplinary tumor board will perform the final clinical assessment and make recommendations based on all information available. Herefore, biopsy and imaging results are unblinded.

However, unblinding is not foreseen at the notional management planning level by the reviewers at any time.

### Data collection and management

#### Plans for assessment and collection of outcomes {18a}

Individual data will be collected electronically and stored in an IT system developed for the administration of this study. Participants who consent to the study will be informed and will receive an “ID number” upon entry into the study. All data will be linked using ID codes to identify the individual. A list of IDs will be kept in a secure location accessible only to the joint principal investigators. Data collection will be both paper and electronic. A summary of the data management will be included in the informed consent form given to participants. An audit trail is used to document data entry and correction.

Training of the investigators is provided prior to the start of the study and for each amendment. The questionnaires used to collect data on treatment planning were developed by the institute itself and have not yet been validated. Standardized, reliable, and validated methods are used for the laboratory tests. All data collection forms are included in the protocol.

#### Plans to promote participant retention and complete follow-up {18b}

All study data collected up to the point of patient withdrawal will be included in the final analysis. Once data are entered into the main database, it will be locked and no further changes are allowed to the original version.

To ensure continuity of study participation and complete follow-up and data collection, regular contact is made once a quarter via email.

Follow-up assessments are ideally conducted face-to-face, but can also be done by phone or mail if needed. We have chosen a time window of 3 months, as this corresponds to the guideline-compliant time window for follow-up of prostate cancer patients in the first 2 years. In addition, this time window accounts for logistical realities and promotes response to follow-up. If a participant from either group is unavailable for the follow-up interview, contact will be repeated by mail three times per measurement point.

#### Data management {19}

Original study forms will be entered and kept on file at the participating site. Participants’ files are stored in a secure and accessible place and manner. Files are stored in numerical order. Moreover, all data will be entered electronically. An audit trail is used to document data entry and correction. Errors will be detected by programs designed to detect missing data or specific errors in the data. These errors will be summarized along with detailed descriptions for each specific problem in data query reports. Access to the study data will be restricted. A password system will be utilized to control access. All reports will be prepared such that no individual subject can be identified. Functions of the database system and programs programmed with the SAS software are used to check completeness, data quality, and plausibility. After the data have been checked, queries are generated and forwarded to the responsible study physician for review and, if necessary, correction. All data will be stored on a secure server at the University of Bonn. The digital files will be stored anonymously in password-protected directories on secure computers. Detailed information on the data management procedures is deposited in the study protocol.

#### Confidentiality {27}

Patient data will be kept confidential and will not be accessible to third parties not involved before, during, and after the trial. All study data will be collected in pseudonymous form. All study-related information will be stored securely at the study site. All participant information will be stored in locked file cabinets in areas with limited access. All laboratory specimens, reports, data collection, process, and administrative forms will be identified by a coded identification number to maintain participant confidentiality. All records that contain names or other personal identifiers, such as locator forms and informed consent forms, will be stored separately from study records identified by code number. All local databases will be secured with password-protected access systems. The study protocol will be granted full public access.

#### Plans for collection, laboratory evaluation, and storage of biological specimens for genetic or molecular analysis in this trial/future use {33}

Within the DEPROMP Trial, physicians will obtain written consent from patients willing to participate in the trial including consent provisions for the collection and use of participant data and biological specimens for current and future genetic or molecular analysis (prostate tissue, blood, urine). The storage of biological samples for genetic and molecular analysis in the DEPROMP Trial takes place in the Biobank of the Medical Faculty of the University of Bonn and the University Hospital Bonn. The University of Bonn has a modern network infrastructure and clear procedures to ensure the confidentiality of the data regarding biological samples.

## Statistical methods

### Statistical methods for primary and secondary outcomes {20a}

The data collected and derived during the course of the trial will generally be presented in individual patient listings (sorted by site) and in corresponding summary tables. The summary tables will be displayed by time of assessment, if appropriate. Continuous variables will be summarized by the following statistics: number of observations, arithmetic mean, median, standard deviation (SD) minimum, and maximum. Summaries of categorical variables will show the absolute and relative frequencies by category and time of assessment. The primary endpoint will be evaluated showing the number and percentage of subjects with changing therapy algorithms due to new information through additional PET-TB together with 95% confidence limits. Secondary endpoints will be summarized descriptively. Sensitivity, specificity, and predictive values will be calculated including 95% confidence limits. Missing data are not replaced by imputation or other measures.

### Interim analyses {21b}

An interim analysis is planned after interventional treatment (underwent PET/MR-TB) of 100 participants. The analysis and report will describe enrollment, treatment adherence, safety and tolerability of treatment of study participants, and efficacy parameters to provide the ethical basis for completing the enrollment of 230 patients. To continue the study, a minimum change rate of 10% in the treatment plan of patients with a tumor, i.e., patients with a prostate cancer detection who received a change in the treatment plan based on the performed PSMA-PET/CT, was established.

The interim analysis will be conducted by the following members of the study team: study director, biometrician, project manager, and monitor/project coordinator.

These are by name:

First study director:

- Name Philipp Krausewitz, MD
  Address Venusberg-Campus 1, 53127 Bonn, Germany
  Institution University Hospital, Urology
  Phone 0228/287- 12610
  E-mail Philipp.krausewitz@ukbonn.de

Second biometrician:

- Name Dr. Robert Németh
  Address Venusberg-Campus 1, 53127 Bonn, Germany
  Institution Study Center (SZB), University Hospital Bonn
  Phone 0228/287–15424
  E-mail robert.nemeth@ukbonn.de

Third project manager:

- Name Dr. Corinna Reineke
  Address Venusberg-Campus 1, 53127 Bonn, Germany
  Institution Study Center (SZB), University Hospital Bonn
  Phone 0228/287–13939
  E-mail corinna.reineke@ukbonn.de

Fourth monitor/project coordinator:

- Name Karina Arnold
  Address Venusberg-Campus 1, 53127 Bonn, Germany
  Institution Study Center (SZB), University Hospital Bonn
  Phone 0228/287–16029
  E-mail karina.arnold@ukbonn.de

### Methods for additional analyses (e.g., subgroup analyses) {20b}

Exploratory subgroup analyses will be performed to investigate the possible differential effects of PSMA PET/CT imaging compared with the current standard of care. However, these analyses are based on the same statistical procedures as those previously mentioned. However, such results should be interpreted with caution because the statistical power for the required interaction effects within relevant regression models is limited.

### Methods in analysis to handle protocol non-adherence and any statistical methods to handle missing data {20c}

Missing data are not replaced by imputation or other measures.

### Plans to give access to the full protocol, participant-level data, and statistical code {31c}

The study protocol will be granted full public access. Summary information on datasets is published in relevant research data repositories and directories. Our data will be kept under lock and key until our main hypothesis-based analyses are completed and published. Three years after the collection of the 2-year postrandomisation follow-up, we will deliver a dataset to an appropriate data archive for sharing purposes. The research team is interested in sharing and pooling metadata with other research groups. Data will not be shared with other sources or used for purposes other than those approved by ethics committees. The principal investigator, in consultation with a data access committee, is responsible for granting access to the data that complies with sponsor regulations. A form must be completed to request access to the data. The principal investigator will review and approve all such requests. Study data will be made available to investigators upon written request specifying the specific objectives and plan of analysis. In the event of conflicts of interest or requests to use data for purposes other than those approved by the Ethics Committee, we will seek advice from the Ethics Committee. Data sharing will be in accordance with the principles outlined in the Good Practice Principles for Sharing Individual Participant Data from Publicly Funded Clinical Trials. External users are bound by appropriate data sharing agreements with the Ethics Committee and sponsor and funder policies.

### Oversight and monitoring

#### Composition of the coordinating center and trial steering committee {5d}

The Coordination Center is composed of the principal investigator, the deputy investigator, and three study nurses. This group will meet once a week to exchange information about the study progress and any problems. These meetings are minuted.

The trial steering committee also includes the study manager, the data management manager, the monitor, and the biometrician. This group will meet after predefined milestones of the study to exchange information about the study’s progress and any problems. These meetings are also minuted.

#### Composition of the data monitoring committee, its role, and reporting structure {21a}

Independent monitoring of the conduct of the DEPROMP Trial will be performed to ensure the safety of participants and the validity and integrity of the study data. Specifically, an independent monitor will conduct a periodic, ongoing review of subject records and regulatory documents including documentation of informed consent, protocol adherence, data collection and quality, source documentation, study management and accountability, adverse events review, and compliance with reporting obligations. The monitoring is performed independently from investigators and the sponsor. Hereby, primary and secondary outcome measures are monitored. Onsite monitoring visits will be performed at least 2 times per year over the course of the study.

Data Monitoring Committee (DMC)

Independent chair

Name Karina Arnold

Address Venusberg-Campus 1, 53127 Bonn, Germany

Institution Study Center (SZB), University Hospital Bonn

Phone 0228/287–16029

E-mail karina.arnold@ukbonn.de

#### Adverse event reporting and harms {22}

The protocol restricts adverse event reporting to predefined adverse events with a Common Terminology Criteria for Adverse Events (CTCAE) grade 3. The survey is performed at study visits 3 (after prostate biopsy) and 4 (1–21 days after prostate biopsy). Details are listed in Table 3.

**Table 3:** Predefined adverse events

Expected adverse events after biopsy:
➢ Hematospermia
➢ Hematuria > 2 days
➢ Rectal bleeding > 2 days
➢ Symptomatic prostatitis
➢ Symptomatic cystitis
➢ Symptomatic epididymitis
➢ Rectal abscess
➢ Urinary retention
➢ Chills in combination with fever > 38.5 °C
➢ Fever > 38.5 °C
Expected serious adverse events include:
➢ Hematuria with urinary bladder tamponade and hospitalization indicated
➢ Urosepsis with hospitalization indicated

#### Frequency and plans for auditing trial conduct {23}

We will review recruitment (and retention) rates on a 4-week cycle to determine the extent of recruitment/retention. The DMC will be informed of the results of these reviews.

#### Plans for communicating important protocol amendments to relevant parties (e.g., trial participants, ethical committees) {25}

Any modifications to the study protocol, which may impact the conduct of the study and potential benefit of the patient or may affect patient safety, including changes in study objectives, study design, sample size, study procedures, or significant administrative aspects, will require a formal amendment to the protocol. Such amendment will be communicated to Ethics Committee at the Medical Faculty of the Rheinische Friedrich-Wilhelms University, Bonn, the Federal Office for Radiation Protection, and the Commission of Clinical Studies of the Medical Faculty of the Rheinische Friedrich-Wilhelms-University Bonn. Protocol amendments will only be implemented after appropriate approval has been received.

#### Dissemination plans {31a}

We expect the results to be published in internationally competitive, high-impact journals. We will present the project and results at local, regional, and international conferences in all relevant disciplines. Additional dissemination will take place via the websites of the University Hospital Bonn as well as the press office.

## Discussion

There is increasing interest in the use of innovative imaging modalities and biomarkers in the context of PCA evaluation to optimize cancer detection and risk stratification. Both MRI and PSMA-PET/CT are integral parts of the clinical routine today. While MRI is currently recommended in the primary staging of biopsy-naïve men, PSMA-PET/CT is used after tumor diagnosis and in advanced stages. The results of the combined use show promising potential. The conduct of PET/MR-TB promises primarily improved assessment of tumor extent and grading and consecutively an optimized risk stratification [16, 17, 24, 25]. Furthermore, PSMA-PET/CT has an impact on the treatment plans of PCA patients in advanced tumor stages [18, 23]. We hypothesized that introducing the additional PET-TB at the biopsy level will not only lead to additional diagnostic yields but also to significant changes in treatment plans. Therefore, additive PSMA-PET/CT-related management adjustments compared with SOC were chosen as the primary endpoint of the DEPROMP Trial. Furthermore, it is not clear to what extent the determination of biomarkers at prostate biopsy will influence our medical practice in the future. Since PTEN and PSMA have been shown to be promising biomarkers for PCA stage and grade, we included the exploratory analysis as a second endpoint in the study.

The DEPROMP study will enable for the first time the evaluation of clinically relevant management changes according to PSMA-PET/CT imaging before prostate biopsy. Despite this, it is unlikely to find a positive influence on the management of healthy men; participants might be reassured by negative findings on high-level medical clarification [26]. Study participation will allow patients to obtain targeted core biopsies of additional PSMA-PET/CT suspicious areas not noticed on MRI, as well as a potential advantage in oncologic therapy by possibly increasing detection of local metastases or local tumor spread. Hence, patients’ morbidity due to over- and underestimation of cancer-related risks will potentially be reduced by an improved prediction of the true tumor grading and extent.

However, some limitations of the study design should be noted: First, using PSMA-PET/CT in a novel instance such as PET-TB and performing it in addition to SOC results in increased costs and examination time for patients, compared to SOC methods alone. Additionally, limited access to this technology and a shortage of trained healthcare professionals must be considered when evaluating its cost-effectiveness. The benefits of incorporating PSMA-PET/CT, or modifying biopsy techniques, may also vary among patient subgroups and may not be generalizable to the entire patient population.

Although the DEPROMP Trial does not include a cost–benefit analysis or patient-reported outcomes, the data collected will provide valuable insights to assess the utility of PSMA-PET/CT as an innovative diagnostic tool for primary PCA. To fully understand the economic and societal implications of PET-TB, it is important to consider not only the monetary costs, but also the changing diagnostic landscape, as well as the patient-level perspective. In this regard, the DEPROMP study will provide results to assess the clinically relevant added value of the innovative and promising PSMA PET/CT technique.

Second, the comparison of MRI only and MRI + PSMA-PET/CT in terms of risk stratification is complicated by the fact that diagnostic workup is increased not only qualitatively but also quantitatively by a larger investigation volume. This bias could be addressed by comparing the performance of SB + MR-TB + abdominal CT and bone scan versus PET/MR-TB. However, because patients are only screened for suspected PCA, the benefit and risk to participants would not balance in this scenario. Thirdly, despite the involvement of highly qualified uropathologists, it should be noted that the histopathologic evaluation conducted within the DEPROMP study is not subject to external review. As such, it is important to take interobserver variability into account during data analysis. Finally, repeatability and transferability to other sites are questionable because examinations are performed in highly specialized tertiary referral centers.

Nevertheless, the DEPROMP study allows prospective analysis of PCA and csPCA detection by each biopsy method used (SB vs. MR-TB vs. PET-TB vs. PET/MR-TB) including performance analysis of corresponding rating systems. It will reveal possible intermethod and pre- and postoperative discordances of tumor stage and grading. Furthermore, the results will allow a comparative analysis of risk stratification by each biopsy method, providing the opportunity to critically assess the need for multiple biopsies and reduce them in the future, if applicable. Last, the determination of the promising biomarker PSMA enables to comprehend correlation and discordance of imaging and pathological staging. Thus, the DEPROMP Trial will make an important contribution to the literature and might take the ongoing debate concerning biopsy pathways to another level.

## Trial status

DEPROMP Trial was initiated in April 2021 and about half of the announced 230 participants have been enrolled by the time of submission. Preliminary analysis of the data (interim analysis of the first 100 participants) indicated no need to adjust the study sample size, as changes in management related to PET-TB were found to be within the estimated range. Currently, the approved protocol version 4.0 and patient information 3.1 are used. Enrollment is expected to be completed within 2023.

## Supplementary Information

**Additional file 1:** **Appendix.**

## Data Availability

Only the members of the study group will have access to the final trial dataset. There are no disclose contractual agreements that limit access for investigators. The data will be kept under lock and key 3 years after the collection of the 2-year postrandomisation follow-up and until the main hypothesis-based analyses are completed and published.

## Abbreviations

PCA: Prostate cancer
csPCA: Clinically significant PCA
nsPCA: Clinically non-significant PCA
PSMA-PET/CT: Gallium-68 prostate-specific membrane antigen positron emission tomography/computed tomography
MRI: Multiparametric magnetic resonance imaging
DEPROMP: DEtection rate of clinically significant PROstate cancer by mpMRI and PSMA-PET/CT fusion biopsy
PET-TB: PSMA-PET/CT guided prostate biopsy
MR-TB: Multiparametric magnetic resonance imaging-guided biopsy
SB: Systematic biopsy
PET/MR-TB: Combined biopsy approach of SB + MR-TB + PET-TB
SOC: Standard of care
ISUP: International Society of Urological Pathology
PSA: Prostate-specific antigen
DRE: Digital rectal examination
US: Transrectal ultrasound
PI-RADS: Prostate Imaging Reporting and Data System
PROMISE: Prostate Cancer Molecular Imaging Standardized Evaluation
CTCAE: Common Terminology Criteria for Adverse Events
PTEN: Phosphatase and tensin homolog
