# Supplementary material for: DEPROMP Trial: the additive value of PSMA-PET/CT-guided biopsy for prostate cancer management in biopsy naïve men—study protocol for a randomized trial
Source: Trials. 2023 Mar 6;24:167. doi: 10.1186/s13063-023-07197-0 (PMC9987083; doi:10.1186/s13063-023-07197-0)
Supplement: Supplementary file 1 — Additional file 1: Appendix. [file 13063_2023_7197_MOESM1_ESM.docx]

**DEPROMP Trial: The additive value of PSMA-PET/CT-guided biopsy for prostate cancer management in biopsy naïve men – study protocol for a randomized controlled trial**

P. Krausewitz^1^, R.A. Bundschuh^2^, F. C. Gaertner ^2^, M. Essler^2^, U. Attenberger^3^, J. Luetkens^3^, G. Kristiansen^4^, M. Muders^4^, C-H. Ohlmann^5^, S. Hauser^1^, J. Ellinger^1^, M. Ritter^1^

^1^University Hospital Bonn, Department of Urology and Pediatric Urology, Bonn, Germany

^2^University Hospital Bonn, Department of Nuclear Medicine, Bonn, Germany

^3^University Hospital Bonn, Department of Diagnostic and Interventional Radiology, Bonn, Germany

^4^University Hospital Bonn, Institute of Pathology, Bonn, Germany

^5^Johanniter Hospital Bonn, Department of Urology, Bonn, Germany

Address for correspondence: Philipp Krausewitz, M.D.

Department of Urology and Pediatric Urology,

University Hospital Bonn

Venusberg Campus 1, 53127

Bonn, Germany

Email: Philipp.krausewitz@ukbonn.de

Tel.: +4915118853551

Orcid-ID 0000-0002-8213-9975

E-mail address of all authors:

- [Philipp.Krausewitz@ukbonn.de](mailto:Philipp.Krausewitz@ukbonn.de)
- [ralph.bundschuh@ukbonn.de](mailto:ralph.bundschuh@ukbonn.de)
- [florian.gaertner@ukbonn.de](mailto:florian.gaertner@ukbonn.de)
- Markus.Essler@ukb.uni-bonn.de
- Ulrike.Attenberger@ukbonn.de
- [julian.luetkens@ukbonn.de](mailto:julian.luetkens@ukbonn.de)
- [glen.kristiansen@ukbonn.de](mailto:glen.kristiansen@ukbonn.de)
- [michael.muders@ukbonn.de](mailto:michael.muders@ukbonn.de)
- [carsten.ohlmann@bn.johanniter-kliniken.de](mailto:carsten.ohlmann@bn.johanniter-kliniken.de)
- [stefan.hauser@ukbonn.de](mailto:stefan.hauser@ukbonn.de)
- [joerg.ellinger@ukbonn.de](mailto:joerg.ellinger@ukbonn.de)
- [mritter@ukbonn.de](mailto:mritter@ukbonn.de)

**Appendix 1**

**A B**

Two different questionnaires are used to conduct patients’ management plans at study visit 4. The fictitious management plans are conducted randomized and blinded by two evaluation teams of two experienced urologists. Each medical evaluator independently proposes treatment plans for each participant: Reviewers of one team receive all PET/MR-TB results and the corresponding questionnaire **A**, while members of the other team receive biopsy results by SOC without PET-TB information and corresponding questionnaire **B**.
